# Therapeutic Touch in the Human Validation Process Model: A Reflexive Thematic Analysis

**DOI:** 10.1111/jmft.70111

**Published:** 2026-01-23

**Authors:** Crystal G. Marroquin, Carson Outler

**Affiliations:** ^1^ Department of Family and Community Medicine University of Texas Southwestern Medical Center Dallas Texas USA; ^2^ Department of Human Development and Family Science Florida State University Tallahassee Florida USA

**Keywords:** experiential therapy, human validation process model, satir, touch

## Abstract

Using touch in therapy is a nuanced and important topic that is especially relevant for experiential therapists. The present study aimed to explore the role and use of touch in Virginia Satir's Human Validation Process Model (HVPM) through phenomenological inquiry. Fifteen participants who use this model were interviewed and asked about their perspective and use of touch in the HVPM. Using qualitative data from expert interviews, the researchers offer best‐practice considerations and examples of if and how touch can be incorporated into experiential therapy. Reflexive thematic analysis was utilized to generate five themes, including: (1) establishing congruent connection, (2) orchestrating touch, (3) handle with care, (4) self of the experiential therapist, and (5) evolving contemporary considerations. Each theme is discussed with the associated codes in addition to direct quotes from participants. These results can inform therapeutic practice and ways to utilize safe and consensual touch for healing in therapy.

The emergence of systemic family therapy brought with it a number of treatment approaches and pioneers from various corners of the world, one of the most eclectic being Virginia Satir, the creator of the Human Validation Process Model. She employed various methods in her clinical work, most notably the use of self and therapeutic physical touch (Satir and Baldwin 1983). Beyond Satir's work, the importance of physical touch in fostering secure attachment bonds has been long recognized (Jakubiak and Feeney 2016). Consequently, the implementation of physical touch in psychotherapy has been understudied, though it may hold value in an environment where a therapist is actively working to create an alliance with clients and intervening meaningfully.

A core foundation of training as a systemic therapist includes instruction on how to create a therapeutic bond with clients through the joining process (Rait 2000). Clinicians early in their careers are taught the risks of violating ethical boundaries that may include inappropriate physical contact. Learning these vital components of practice while simultaneously being introduced to a model that promotes the use of self and touch may lead those curious about this approach to focus on a more popular or manualized approach that includes solely verbal interventions. Touch is a dynamic intervention that can manifest in more forms than just therapist to client contact, such as the facilitation of touch or the manipulation of the therapeutic space to create connection. When it comes to physical intervention, training therapists must be taught the when and how in addition to the right and wrong.

As the practice of experiential therapy grows and more clinicians integrate the HVPM into their approach, it is essential to critically assess the role of touch in therapeutic practice. The purpose of this study was to explore how experiential therapists use and facilitate touch in therapy, while also presenting evolved conceptualizations of touch within the Human Validation Process Model.

## 1 Literature Review

### 1.1 Core Tenets of the HVPM

The Human Validation Process emphasizes the genuine value, respect, and appreciation for the miracle and goodness of all people (Haber 2002). This deeply held virtue impacts both the concepts and practice of this model. This model has several core tenets including the beliefs that congruence between internal feelings and external behaviors is essential, self‐esteem is at the core of functional and dysfunctional interpersonal processes, and that overt and authentic communication is essential for relationship health (Erker 2017). Congruence is a multifaceted construct in this model related to communication and harmony (Satir and Baldwin 1983). In the HVPM, congruent communication is often described as “saying it straight” or saying what one means and doing what one says (Englander‐Golden and Satir 1990). This is an overarching goal for clients, and therapists who practice this model should also strive for, and model congruence. In addition, this model asserts that self‐esteem affects individual and systemic functioning, for better or worse. When self‐esteem is low and threatened, people will protect it by placating, blaming, being ultra‐reasonable, or distracting from the exchange (Satir 1972). The HVPM aims to help clients improve their self‐esteem, become more congruent, and communicate directly. The HVPM also focuses on noticing, naming, and modifying communication patterns within the system. Satir highlighted that communication is the exchange and making of meaning between people and includes all five senses (Satir 1976; Satir and Baldwin 1983). The HVPM encourages therapists to help clients better understand the somatic responses associated with positive and negative stimuli to support a mind‐body connection (Satir et al. 1991). To achieve these goals therapists can utilize a variety of tools, such as metaphor, sculpting, humor, use of self, and touch.

### 1.2 Touch in the HVPM

It is first necessary to highlight that any physical engagement with a client must be consensual, informed, and intentional (Pinson 2002). Before engaging in touch with the client, the therapist must consider the client's identity, clinical history, and the current therapeutic alliance (Phelan 2009). Touch in the context of this model may include the therapist putting their hand on the client's back in an act of soothing or offering a hug when the client feels overwhelmed. Satir believed that physical touch was a method of connecting with clients to help them feel safe and grounded in a therapy session, but that it must be done with purpose (Satir and Baldwin 1983). Early teachings and videos of this model depict Satir engaging with clients heavily through physical touch, in the form of holding clients' hands, faces, and shoulders. However, for different reasons, this may not be congruent for all clinicians, and the extent of physical touch with clients that feels authentic in the therapeutic space may vary.

In Satir's popular text *Step by Step*, the authors highlighted, “…that her touch is not an automatic, mechanistic ‘technique’ that she uses indiscriminately. She was very sensitive to the cues she receives about people's boundaries and will not transgress them” (Satir and Baldwin 1983). Touch in the HVPM is deliberate and intentional as a form of soothing or intensifying a moment or experience. For example, one might sit next to a client and put their hand on the client's back to provide comfort if they are feeling overwhelmed while processing a difficult topic. In addition, the therapist might physically lean in or place a hand on the client's arm or knee when engaging in deep processing to show physical support while also aiming to ground the client in the conversation.

As previously mentioned, touch, within the context of this model, may not always be physical contact between therapist and client. In addition to physical touch, there is the facilitation of touch, where the therapist encourages relational clients to hold hands with each other or move closer to one another. Therapists who practice this model may never engage in physical touch with their clients but instead facilitate an experience of touch among couples or families. With an individual, the therapist might encourage the client to offer themselves comfort in the form of a hug or relief of a somatic response through massaging the point of stress on their body. As this model highlights the importance of helping clients recognize and acknowledge their internal responses in addition to their external ones, facilitating touch as an exploratory tool may be beneficial. In systems that include more than one person, touch may be incorporated as a way to create new or resemble previous forms of closeness and intimacy as desired by clients. For example, the therapist could instruct one partner of a couple to hold their partner's hand or place an arm around their partner as they discuss a difficult topic or experience. In addition, touch may occur when implementing other interventions central to this model, including a family, sculpt, where the therapist may position clients using their hands. Although a component of the HVPM, other systemic models do not incorporate or encourage physical engagement with clients, leading to curiosities surrounding the utility of touch in treatment.

### 1.3 The Polarization of Touch

The use of touch in therapy, regardless of the therapeutic model, is a nuanced and debated topic. Although many therapists may not purposefully use touch in their work, past research has found that most therapists do touch their clients situationally (Hunter and Struve 1998; Pope et al. 2006). A qualitative study on clinical psychologists' use of touch in therapy found that deciding to touch a client was typically instinctual (Harrison et al. 2012). Therapists may use touch for a variety of reasons, such as to greet, console, and encourage clients, or to restrain a violent client (Rovers et al. 2017). However, many therapists are hesitant to discuss any use of touch for fear of allegations of wrongdoing or suspicion of misconduct (Stenzel and Rupert 2004).

Proponents of touch in therapy suggest that touch in itself is therapeutic (Berendsen 2017). Touch is a pre‐programmed language of the human experience that is essential to our development (Gothard and Fuglevand 2021). A recent systematic review and multilevel meta‐analysis by Packheiser et al. (2024) synthesized findings from 137 empirical studies and 75 broader systematic reviews. The study reports medium‐sized overall effects of touch interventions on physical and mental health in adults. Specifically, they discovered that touch interventions significantly reduced depressive, anxious, and pain symptoms; moderator analyses showed that clinical samples had greater mental health benefits when compared to normative samples (Packheiser et al. 2024). Specific to therapy, advocates of touch highlight how touch can help to enable containment and security (Courtney and Gray 2014; Pinson 2002) and facilitate communication (Salzmann‐Erikson and Eriksson 2005). Furthermore, touch can help clients utilize their somatic knowledge and reconnect their minds with their bodies. Research in neuroscience supports this mind‐body connection and has shown that body psychotherapy can help treat chronic depression and trauma (Langmuir et al. 2012; Leitch et al. 2009; Röhricht et al. 2013). Prior research has also suggested that touch is most effective in therapy when there is an open dialog about it (Westland 2011). Many argue that touch should be a foundational topic in master's programs with opportunities to practice using attuned, safe, intentional, contextual, and consensual touch (Wright 2020).

On the other hand, therapists may feel uncomfortable or unsure about touching their clients for various reasons. Most counseling and therapy codes of ethics do not directly address touch, and few ethical decision‐making models consider touch (Calmes et al. 2013). To be clear, sexual or violent touch is considered unethical in every discipline. However, many training programs provide inadequate training related to therapeutic touch (Wright 2020), which may lead to misunderstandings regarding whether touch is appropriate. Furthermore, the debate regarding touch has remained dichotomous, disregarding the how and when of using touch with clients (Stenzel and Rupert 2004). For many therapists, the decision to avoid touch may be rooted in risk management or uncertainty about how to integrate touch into trauma‐informed care. Understandably, many supervisors may discourage their supervisees from using touch to avoid potential ethical or legal issues. Touch may be viewed as risky, especially if clients misinterpret it (Glickauf‐Hughes and Chance 1998). Lastly, modern social movements have pushed for more attunement to power, autonomy, and trauma. Therapists may be concerned about their touch being experienced as harmful, violating, or retraumatizing (Calmes et al. 2013). Without proper training, many therapists feel ill‐prepared regarding the use of therapeutic touch, consequently erring on the side of caution and avoiding touch altogether.

## 2 Current Study

Interventions within the HVPM, especially therapeutic touch, must be implemented with intention, curiosity, and overt ethical caution. Although touch is often discussed in descriptions of experiential approaches, the literature lacks a clear account of how this intervention has evolved within the context of Satir's model. These progressed conceptualizations also require consideration of the nuances experienced by modern‐day clients. In the current study, the researchers worked to better understand how practitioners might use or facilitate touch and their perceptions of its importance in relation to the HVPM and their clinical work in general. Since the model's inception in the late 20th century, social norms around touch and connection may have changed. Thus, the researchers were interested in the participants' implementation and conceptualization of touch in therapy in today's climate, and in how they have not only adapted to the needs of present‐day clients but also made touch their own. This study aimed to answer the following research question: How do systemic therapists informed by the Human Validation Process Model make meaning of and conceptualize the clinical use of touch?

## 3 Methods

### 3.1 Positioning and Paradigm

The authors would like to acknowledge their social locations in the name of transparency and self‐reflexivity. Both authors use the HVPM in their clinical practice. They identify as able‐bodied, cisgender females who are early in their careers as marriage and family therapists. They both hold master's degrees and with one author holding a PhD and the other pursuing their PhD. The first author is Hispanic, and the second author is white. We want to acknowledge that our unique intersectional identities influence our use of touch in therapy and this research. In discussing identity and power, it is important to explicitly address how our bodies hold and are intertwined with our lived experiences and trauma. When discussing touch in therapy, the literal physicality of our human forms, including their shape, size, skin color, age, and gender expression, is also central. For example, our bodies, as we showed up in the interviews with our participants or just as we show up in therapy with our clients, are not neutral instruments. Touch in therapy is shaped by perceptions of femininity, masculinity, race, age, ability, and normative versus divergent appearances. Due to gender socialization and norms in the United States, our use of touch may be perceived as nurturing, less threatening, and more socially acceptable. Throughout the data analysis process, there was intentional discussion about how the researchers' identities, bodies, and clinical perspectives informed the results of this study.

In addition, this work is situated within a constructivist‐interpretivist paradigm, grounded in a relativist ontology and a constructivist epistemology. A relativist ontological stance assumes that reality is multiple, subjective, and shaped by context (Guba and Lincoln 1994). From this lens, there is no single objective reality of touch. Our constructivist epistemology asserts that knowledge is co‐constructed through dialog (Creswell and Poth 2018). In our work, the co‐constructed meaning between participants and researchers was not aimed at producing universal truths but instead contextual reflections. This paradigm acknowledges the situated and interpretive nature of qualitative research and aligns with our goal to capture therapists' meaning‐making processes about touch (Guba and Lincoln 1994). In designing this study, we prioritized methodological integrity by ensuring both fidelity to the participants' lived experiences and utility in achieving our research goal to generate meaningful contributions to HVPM theory (Levitt et al. 2017).

### 3.2 Methodology

Guided by our research question and the recurring theme of nuance and context in existing touch research, phenomenology was well‐suited to elicit rich and descriptive accounts of the participants' experiences. Phenomenology emphasizes first‐person accounts with the aim of uncovering the essence of a phenomenon (Van Maanen 1998). Grounding this study in a phenomenological approach provided clear alignment with our relativist ontology and constructivist epistemology (Creswell and Poth 2018). From this standpoint, the meaning and collective experience of touch in the HVPM are understood as subjective and conditioned by the personhood of the therapist and client. To understand how touch is experienced, perceived, interpreted, and implemented, participant narratives are prioritized. Thus, phenomenology best informed the generation of knowledge from participants' lived experiences, capturing the nuance necessary to interpret the complexities of touch in clinical practice.

### 3.3 Method

To analyze our data, we employed a reflexive thematic analysis, which allowed us to expand the existing HVPM theory while attending to participants' lived perspectives and insights (Braun and Clarke 2025; Creswell and Poth 2018). Reflective thematic analysis is a qualitative method used to identify, analyze, and interpret patterns that exist within the data (Braun et al. 2022) with a focus on the role of the researcher. This method acknowledges subjectivity and encourages ongoing reflection throughout the data analysis to acknowledge how the researcher's perspective can shape the themes that emerge. A deductive approach was utilized, as the coding and theme development were guided by existing ideas related to the HVPM (Braun and Clarke 2022b). The “fit” among our design, method, and research question was evaluated for alignment (Braun and Clarke 2022a; Willig 2013). Our reflexive thematic analysis was experientially oriented and well‐suited to our research question, as it focused on people's practices and behaviors. This approach can capture patterns across organizing themes, be integrated with a variety of theoretical frameworks, and address questions related to people's experiences (Braun and Clarke 2022a, 2022b). The flexibility of reflexive thematic analysis allows for a nuanced understanding of the diverse ways therapists incorporate touch, while its focus on the researcher's perspective ensures that the analysis remains sensitive to the complex, subjective experiences inherent in therapeutic practices.

### 3.4 First Wave Data Collection

#### 3.4.1 Participant Recruitment

The data for the present study were collected in two waves. The first wave of data collection was conducted as a part of a larger study aiming to create a conceptual framework for the HVPM (Outler and Marroquin 2025). Potential participants were considered experts if they had a robust clinical, professional, and/or scholarly dedication to and mastery of the Human Validation Process Model. Potential experts were contacted via email.

#### 3.4.2 Procedures

A description of the study, survey link, and informed consent were shared with potential study participants. The survey included various measures and open‐ended questions assessing participants' use and understanding of the HVPM. Those interested completed the survey and were given the opportunity to opt into an interview with the primary researcher. Participants who opted into the interview portion were scheduled to meet with the primary researcher via Zoom. They were asked about their experiences learning about and using the HVPM in addition to their understanding of the core concepts and interventions. Interviews in the first wave of data collection included one question about the use of touch in therapy in addition to one probe. Participants were asked: how do you use or facilitate touch with clients? If they did use touch in their work, they were then asked: Are there any considerations you keep in mind when using touch? The current study aimed to capture a wide range of experiences, and upon completion of the first 10 interviews, there was not sufficient information power (Malterud et al. 2016) therefore five additional interviews were conducted. Information power refers to the amount of information a sample holds that is needed to address the study aims (Braun and Clarke 2021).

### 3.5 Second Wave Data Collection

#### 3.5.1 Participant Recruitment

In a second wave of data collection, the sample was expanded to include a specific focus on the use of touch. These participants were recruited using snowball sampling. No compensation was provided for participation in the study. Study procedures were overseen by the University's Institutional Review Board.

#### 3.5.2 Procedure

Interested participants were asked to complete a brief survey, including the consent and demographic questionnaire, before scheduling an interview with the primary researcher. Once scheduled, participants completed a 1‐h recorded Zoom interview with the primary researcher. Using a semi‐structured interview guide that focused on the touch intervention, the researchers asked participants about their perception and use of touch using this model. Sample questions included, “As we know, Satir's model highlights using touch. How do you, personally, use or facilitate touch with clients?” and “Do you think touch being an intervention in this model draws therapists in or pushes them away from the HVPM?”. Participants were encouraged to share as much or as little as they were comfortable with regarding their use of touch when using the HVPM. Further questions inquired about how participants' identities played a part in their decision to incorporate touch in their practice. All participant data were deidentified and saved in a secure OneDrive file. Interviews were transcribed verbatim using the software service Otter.ai and checked for accuracy.

### 3.6 Complete Sample Demographic Characteristics

The final study sample includes 15 interview participants. Years of experience using the HVPM ranged from 7 to 60 (*M* = 26.3, SD = 16.9). The sample included nine women and six men. Participant ages ranged from 33 to 79 (*M* = 56.4, SD = 17.9). The self‐reported racial/ethnic breakdown was 10 white non‐Hispanic participants, 2 Hispanic participants, 1 Black participant, and 2 participants who selected “other, non‐Hispanic.” Five of the participants were both serving in faculty roles and practicing clinical work, five were only in clinical roles, three were in other professional roles, one was exclusively in a faculty role, and one participant did not disclose their current professional role. All participants were currently living in either the United States or Canada. For the 10 clinically active participants, the majority of them were in private practice settings serving individuals, couples, and families, with some participants also having experience in medical settings.

### 3.7 Data Analysis

Reflexive Thematic Analysis is a method of systematically organizing qualitative data to bring conceptualizations and ideas to the surface in the form of significant codes and themes (Braun and Clarke 2022a). This iterative process allows researchers to become closely familiar with what the data says, working through six stages of review in which each experience with the data makes the meaning clearer (Mihas 2023). Throughout this organic analytic process, researchers used note writing at the construct level to capture impressions, refine codes, and develop themes.

In the first stage, we both worked to familiarize ourselves with the data by reading each of the participant transcripts individually back‐to‐back to become familiar with the unique perspective of each participant. One example of a stage one note read, “I can tell this is an older therapist and they sound really confident in using touch in therapy. What does this mean for newer therapists who may not ‘emulate’ a similar energy to the model founder who was an older woman?” These were the types of reflections that were discussed among researchers to explore how each perspective might impact the individual coding processes. The second stage consisted of the initial generation of codes, which was done by both researchers individually before coming together to discuss. Sample codes included “touch as use of self” and “descriptions of use of touch in practice”. Through rounds of coding, our codes were reflexively refined. For example, the initial code “types of touch” was further refined to codes such as “grounding touch” and “evocative touch”, and evolved into the final codes “soothe and comfort” and “amplifying the moment.” This was followed by our third stage, the preliminary generation of themes which included the organization of codes and the exploration of potential patterns in the data. As practitioners who have utilized and supervised clinicians using the HVPM, we explored how our own subjective realities may have influenced interpretations of the data. The initial theme “indirect use of touch” represented a broad, generic cluster and evolved into “orchestrating touch”. This refined theme highlights our shift toward an interpretive, meaning‐based pattern (Braun and Clarke 2022a). Stage four consisted of the development and evaluation of themes. We worked to explore how our individual perspectives and clinical orientations might have influenced our decisions surrounding what constitutes a theme. This led us into stage five, where we defined themes with representative names. The sixth stage consisted of finalizing our themes and presenting the interpretations of our data extracts (Braun and Clarke 2022a).

### 3.8 Trustworthiness and Credibility

Trustworthiness in qualitative research refers to the techniques used to maintain rigor, transparency, and credibility throughout the analytic process (Ahmed 2024; Creswell and Poth 2018). In line with the recent reporting guidance, we followed Braun and Clarke's (2024) Reflexive Thematic Analysis Reporting Guidelines, which emphasize transparency, congruence, and reflexivity as key markers of trustworthiness in reflexive thematic analysis. These guidelines (Braun and Clarke 2024) reinforce that trustworthiness in reflexive thematic analysis is achieved through sustained reflexive engagement rather than adherence to a checklist. Throughout this research, we implemented multiple reflective practices. As therapists trained in the HVPM, we had ongoing discussions about how our clinical and personal experiences and assumptions about touch might shape our work. For example, in early interviews, we assumed that all participants who used this model and expressed interest in the study would utilize touch. When this assumption was not supported, we critically examined how this expectation could have influenced our perception of participants' accounts and actively bracketed its impact in code and theme development (Tufford and Newman 2012).

Credibility, one of the pillars of trustworthiness, refers to the extent that findings are faithful to participants' accounts (Korstjens and Moser 2018). In reflexive thematic analysis, credibility is achieved through careful attention to the interpretive role of the researcher (Braun and Clarke 2022b, 2024). Credibility in this study was supported through detailed reflexive note‐writing, peer debriefing discussions, maintenance of an audit trail of coding decisions, and engagement with multiple data sources to enrich interpretation (Nowell et al. 2017). Together, this ongoing reflexive practice helped support the confirmability and dependability of the results.

## 4 Results

The deductive reflexive thematic analysis of participant interviews led to the refinement and curation of five themes and 14 codes that captured both theoretical constructs and the lived experience of participants. The overarching themes generated were (1) establishing a congruent connection, (2) orchestrating touch, (3) handling with care, (4) self of the experiential therapist, and (5) evolving contemporary considerations. Each theme will be discussed with the associated codes in addition to direct quotes from participants that support each theme.

### 4.1 Establishing Congruent Connection

In the Human Validation Process Model, “making contact” is the process of creating a genuine connection with and between clients, where information can be shared safely, and a sense of hope for the future is engendered (Satir 1976). Participants overwhelmingly conceptualized touch as the physical connection between themselves and their clients. They cited several purposes for using touch in the therapeutic setting, which are expounded in the codes (1) soothe and comfort, (2) interpreting routine rituals, and (3) amplifying the moment. Despite the intention of said physical contact, participants noted the importance of explaining the purpose of touch. From the beginning, it can be helpful to explain to clients that touch is a part of this model and how it may be used, with consent, to aid in healing throughout sessions.

#### 4.1.1 Soothe and Comfort

Touch was often viewed as a tool to help clients who are in a heightened emotional state to reorient themselves to the present. One therapist explained,

In my work with trauma, there have been situations where I've seen people become dissociated, or kind of outside their window of tolerance in a state of fight or flight, and with their permission, because I don't want to re‐traumatize them, sometimes touch is grounding that way. Helping bring people back to reality like, ‘Hey, are you okay if I just put my hand right here on your knee for a second while you while you do this breathing? To kind of bring you back’.

Another participant shared, “If it's in the right time and the right place you know, feeling the hand when somebody's having a terrifying, terrifying memory. [Saying] ‘I'm here. You're not there. We're together now”. In addition, some participants highlighted the differences between touch that felt normal for them to use outside of therapy versus touch that was more unique to the therapeutic setting and early representations of the HVPM. One participant shared,

…. touch for me, like to do it and incorporate it in the use of the model is not a natural thing for me. But there have been times I wanted to do it in session…for example I had a client whose boyfriend overdosed when I was working in inpatient, and it felt really normal for me to pat her back.

#### 4.1.2 Interpreting Routine Rituals

Several participants also discussed how clients often seek touch as a normative greeting for salutations. This social and cultural signal is often commonplace in other spaces of community life (e.g., workplace, church, medical settings) and family life, where hugs can be a way to connect with a loved one. One participant stated,

I have many, many clients who just want to give me a big hug at the end of the session like it's a very common thing. Some do and some don't. I take it by their cues, you know, I don't impose it. But it's because it's such a way of deep connection, that touch can feel very kind of affirming of that…

Some participants also noted that it is imperative to attune to cues from the client along with one's own comfort with touching. One therapist shared, “When you're leaving therapy, and [clients are] like, ‘do I get to hug you now?’ or things of that nature, just very genuine and not in a creepy way… I always say, ‘I'll settle for a handshake,’ or depending on the client, ‘Yes, of course’.”

#### 4.1.3 Amplifying the Moment

Finally, a code arose of touch being used to heighten or intensify an emotional experience in therapy. Participants often reflected on using touch with couples or families to exemplify a process or connect loved ones in a tense exchange. One therapist explained,

When my clients [have] a little bit more of their guards down or are a little bit closer, I will joke with them, and I'll be like, ‘Well, what's this about?’ and get them to notice what might be, and sometimes it's touching… then I'll ask them how they can rely on that to help them through tensions or miscommunications.

Many participants described touch as a tool to increase the depth or power of a point in the therapy room. One participant shared, “[touch is] turning up that emotional intensity, or that emotional anxiety, right? Like bringing that intensity to the moment”. Another participant similarly noted that with touch, they “want to be able to amplify an emotional moment.”

### 4.2 Orchestrating Touch

Participants in this study did not always denote touch as an intervention that was inherently direct, but at times something that was delivered through essence or closeness to the client, in addition to the facilitation of touch or highlighting client touch that occurred without being prompted. This theme incorporates three codes: (1) manipulating the space, (2) facilitating connection, and (3) spotlighting natural client touch.

#### 4.2.1 Manipulating the Space

Participants noted that at times it felt more authentic for them to be closer to a client when discussing a hard topic or when they wanted to encourage both themselves and the client to be more present. One participant described their conceptualization of this process and shared,

I believe this is what the Satir model is really kind of calling for when it talks about touch is more about that closeness, exhibiting that, like ‘I'm here, you can trust me’. It's less about the touch itself in my eyes, and more about just how can I physically offer that I'm close, that I'm here, and that I'm supportive.

They went on to share a specific example of how they might engage with the client and the therapeutic space in a way that does not include them physically touching the client. They highlighted that,

It's kind of a proxy closeness of manipulating and using the room to be able to help facilitate close closeness with clients, between clients, etc. Whether that's a hand on the arm of the chair, whether that's stepping a little bit closer, whether that's moving your chair a little bit closer, or moving the chair to the side, so it's less confrontational and more like, ‘Hey, we're in this together’ type deal. That is a like I said, I kind of call it a quasi‐touch, where we're still using that spirit of closeness and connection, without having to break that physical touch barrier that you and your client might not be comfortable with.

Participants also noted that Satir's method of introducing one's presence to the space was useful when beginning to work with new clients. For example, another participant highlighted, “what [Satir] did with new people where she didn't have that [touch as an intervention/consent] established, she would go like put her hand there [near the client], she wouldn't touch, she would put her hand in the field.”

#### 4.2.2 Facilitating Connection

In addition to manipulating the therapeutic space, participants noted the value of creating moments of connection through touch for relational clients, which was utilized to “amplify an emotional moment”. For example, one clinician shared that they often do this physically and verbally by, “joining their [clients] hands” or asking, “‘can you guys take each other's hands, can you sit next to each other?’” Participants suggested these moments allowed for clients to begin building habits of closeness starting in therapy in addition to facilitating physical support between them during difficult conversation. There was also discussion surrounding the value of taking steps towards physical connection by starting with other methods of connection, for example, one participant stated:

I don't always begin with a ‘hold each other's hand’, you know, I begin with eye contact. I see it as a connective moment and so we need to do something actionable about that. That's either, like, look at each other, or for some people that's hand holding or getting closer on the couch.

Another participant emphasized that creating the opportunity for connective moments before or after the session opened the door for meaningful rituals to occur in the therapeutic space. They emphasized,

…having clients hug upon arrival, or when leaving, you know, facilitating touches is an important part of how families relate, and all this goes back into the human elements of things, and isn't based on like, what society has deemed as appropriate or not appropriate.

#### 4.2.3 Spotlighting Natural Client Touch

In addition to encouraging the continuation of connective moments, the importance of noting when clients began seeking out touch from their loved one unprompted was expressed as well. Once clinician did this by using humor and noting when:

…they're letting their guards down or sitting a little bit closer. I will joke with them, and I'll be like, ‘well, what's this about?’, you know and get them to kind of notice the touching and warmth and I'll highlight it for them.

This was also used to showcase the client's strengths in connection to their therapy goals. This clinician went on to describe the importance of noting that touch is a “strength they're coming into therapy with. I have some couples who that's natural to them to have that touch with each other. Then I'll ask them how they can rely on that to help them through tensions or miscommunications.” In addition, participants shared that it was valuable to process the experience of a natural touch and the impact it had on the present moment to reinforce the behavior when the impact is positive. One participant did this by noticing,

When natural touch is occurring, if they're talking and one reaches over and takes the other one's hand, we're going to stop and we're going to highlight that. We're going to talk about it and we're going to process like ‘you just grabbed his hand, tell me what you were feeling and why you did that. What was your experience like when she did that, and what does it feel like right now as she's holding your hand?’

To be an experiential therapist is to be aware of all parts of the client experience, including their physical sensations and processes in the therapy room. Participants highlighted the value of incorporating a more indirect form of touch but reiterated that all methods of connection come with necessary considerations for client safety.

### 4.3 Handle With Care

Another salient theme for participants was conceptualizing and ensuring trauma‐informed and ethical use of touch with clients. Specifically, the therapists reflected on how clients' trauma history might impact their comfort with giving or receiving touch. To encapsulate these reflections, the codes of (1) ethics in practice and (2) process‐oriented consent emerged from the data.

#### 4.3.1 Ethics in Practice

Participants acknowledged the current landscape of touch in the therapeutic setting, although they differed in their reactions on how to approach it. Participants shared concerns about their touch being misinterpreted and a looming concern of legal ramifications. One participant shared,

Consent and permission is important because especially if the nature of the trauma was physical in nature, then to reach out and just touch them could have a counter effect. I guess with children, I'm a little more hesitant too. I think children need it. But I as a clinician, ethically I'm worried about like ‐ I don't want anyone to, to perceive it in a way that it was not meant, which can happen. So, I'm careful about touching children, maybe even more so than consenting adults. But I still think there's a place for it.

Another participant described,

I think the current context of therapy… the idea of touch in the professional space has people kind of on guard about liability, number one, and two, because we have so many public examples of people harming other people institutionally…I think people even buckle at the idea of hugging their clients, even after many sessions, you know. I think it's just the sort of professional cultural climate these days. Most people are pushed away from [the model], when they see that [touch] is an element.

Participants also named their hope to move away from black‐and‐white thinking regarding touch in therapy. Rather, they acknowledged the need for ethical decision‐making to be guided by context‐specific interactions. For example, one clinician remarked,

These days, touching is sort of out of the picture with most other models…you have to be very careful about touching clients…there are people that abuse touch. A lot of therapists can abuse touch. And that's a problem, but to just say, okay, no touching. I don't think that's the right solution.

One participant highlighted not only the considerations that affect the use of touch but also the role of the therapist in making that decision. They suggested,

Considering ethics, considering the personal story of the client, and then considering the clinician comfortability, some therapists from my experience, and even training, don't necessarily feel comfortable hugging their clients. Others feel like you know, certain moments, it is appropriate, especially if their client is in high distress, or in tears.

They went on to say, “Thinking about, depending on what they've been through, touch may not necessarily feel welcomed, or seen as comforting.”

#### 4.3.2 Process Oriented Consent

Participants reiterated the need for clients to give explicit and ongoing consent for touch beyond the informed consent in the intake session. Clinicians conceptualized consent as an ongoing process that offers the client the option to revoke their original yes at any point. One participant said,

Whether it be touch, whether it be an intervention, just because the client consented via the paperwork doesn't mean that it's just a pass for the therapist to do whatever they want…If we're doing something inclusive of touch that we're not seeing eye to eye with, and you're not comfortable and consenting to, then we're not going to do it.

They went on to further explain,

Consent is fluid, you know, like, it's not something like you say, yes, once means you have to say yes, all the time…If we create an environment where [clients] are able to grasp their own consent and agency over that moment that touch is involved? It's more liberating, because it's connected to the other part of the model, right, their self‐concept. They want to do it. They're inviting it in. It's scary, but they're inviting it in.

Participants also shared *how* they ask for consent when using touch in the HVPM. One participant shared,

Asking, you know, ‘is this, okay’? You know, ‘is that okay’? Would it be okay if I touched your hand like this? Or would it be okay, if I gave you a hug, or, you know, something like that. And to back off if they say ‘no', I don't, I can't do that right now.

Similarly, another participant also highlighted how getting affirmative consent for touch is not only ethical but also can help to model for clients. They said,

Even if I'm putting a hand on a wrist…'Hey, can I put my hand just right here?' I see that as really important. And I see that also as a way of modeling, I think, healthy connection and relationships. Just because one partner wants to touch another partner doesn't mean that it's always okay. Just because you're married doesn't mean that it is unilateral, constant consent. I think asking and inquiring and then playing it by ear each time is really, really important.

### 4.4 Self of the Experiential Therapist

The Human Validation Process Model is unique from other systemic therapy models in that it aims to support and utilize the therapist's personality and way of being when intervening (Satir and Baldwin 1983). With this, it is imperative that the therapist develop a critical awareness of how their identity, presence, and personality affect the use and potential impact of an intervention in therapy, especially when considering the use of touch. Within this theme, the two codes (1) personhood and (2) clinical congruence were explored.

#### 4.4.1 Personhood

One participant emphasized how specifically their gender identity and age are at the forefront of their decision‐making process when considering touch. They shared,

As it relates to dimensions of my identity I believe that gender plays a large part, that is, I'm always aware of how myself as a man, and how touch can be perceived by anybody else. I also understand that, you know, many folks that sit in the couches in our rooms have had, particularly men in their lives that have not practiced good touch, whether it be abuse, manipulation, things of that nature. I'm always aware of that and how that plays a part.

They went on to add,

Another part of my identity that I'm always aware of is my age. Particularly as it relates to working with, with youth…I do not practice touch in the room beyond, say, sculpting with a family and, and helping, you know, a teenager, if you will kind of move to specific places or modeling in that regards.

Another participant comically noted that the model founder, Virginia Satir, had a physical presence that played a large role in the delivery and reception of a touch intervention and noted their perspective when they shared,

Here's the caveat. You know, Satir had like this BGE, the ‘Big Grandma Energy’, right? And she could just, she could just do it [utilize touch] in a way that was not threatening. I'm a straight white dude that can be perceived as upper class… so I can't just go in for touch the same way Satir could.

#### 4.4.2 Clinical Congruence

In addition to understanding the role of identity and presence, it is important for therapist to have the understanding that they can use touch if it aligns with their personhood and comfort level, but should not feel pressured to implement this intervention. There may be discomfort or fears that arise when considering this intervention, and all should be explored before implementation. For example, one participant shared that direct physical touch did not align with their cultural background and said, “I'm Hispanic, and we're not really touching people and maybe that's just my Hispanic family…and that's not a natural thing for me to do. I just like to keep those boundaries.” In addition, participants outlined different fears concerning how touch might be received. One participant shared,

It's kind of it's a vulnerable thing to like, reach out to someone, because what if they like slap your arm away? How do you repair that, right? It's one thing to say something like, ‘I'm proud of you’, and they're like, ‘whatever’, and blow it off. That doesn't create room for so much of a rupture, right? But if I reach my hand out, and I try to, like, embrace you or something, that's more vulnerable to me. I'm taking my own risks in doing that. I had to become comfortable taking those risks and knowing when it's appropriate to take that risk.

In the process of developing one's clinical approach, there are essential reflexive components that must come into play to explore the way a clinician wants to exist in the therapy room. An experiential clinical approach can evolve over time to incorporate touch as long as the proper considerations are taken into account.

### 4.5 Evolving Contemporary Considerations

As clinical approaches continue to evolve to meet the needs of modern‐day clients, the model concepts and interventions must be understood through a contemporary lens. Participants in this study emphasized the importance of considering the more nuanced and invisible aspects of utilizing touch ethically and systemically in therapy. The final theme that was generated from the data consists of four codes, including (1) cultural considerations, (2) power dynamics, (3) therapeutic setting, and (4) model evolution.

#### 4.5.1 Cultural Conceptualizations

Participants in the current study outlined the importance of taking time to understand the client's cultural context and how that may impact the implementation of an intervention in therapy, especially touch. One participant shared the value of,

…thinking through that socio cultural attunement. I am in South Florida, and we have a lot of diverse families, from the Caribbean, many who are also Hispanic. Many have explained to me that they are very expressive, and they welcome touch and connection, in terms of celebrating. It can be seen as socially inappropriate or offensive, if you don't welcome someone in a session with a hug or offer a touch of just signaling that you're here with them and that this is a safe space. It shows them that you care not only about what they're sharing, but also what's important to their cultural heritage and social norms. I think that really informs how I practice and being able to also not assume and learn with the client, even if they are of a Caribbean background, Haitian, Bohemian, Jamaican.

In addition, some participants discussed the differences regarding touch, and from the perspective of collectivist versus individualistic cultures. One noted that, “the collective spirit and the collective engagement of gathering for the sake of physically being around each other and spending time together. It's something I think is fundamentally part of a lot of collectivist cultures.” Understanding the client's values surrounding closeness and support can help guide the therapist's clinical judgment when considering the utility of touch with their clients.

#### 4.5.2 Power Dynamics

Another component that participants emphasized as being vital to their decision‐making when incorporating touch in therapy was the consideration of power dynamics. They included how current events and social movements related to bodily autonomy impacted their views and conceptualizations of touch when utilizing this intervention. One participant noted,

What comes to my mind is the #MeToo movement, as well as George Floyd and Black Lives Matter. We have to consider these social movements with touch. You know, Satir, as a person and as a model… although it is, client centered, person‐centered, the therapist is very active, engaged in system understands their power, and uses their power mindfully.

Another participant highlighted how the therapist must work to be overt in ensuring the client is aware of their autonomy as a client and noted, “it's important as it relates to Black Lives and making sure that like the client understands, feels, knows, and is respected to be in full control in the therapy room.” Therapists also highlighted how there may be invisible pressure to engage with the therapist approach and potential use of touch considering the position and privilege of the therapist in their role. One participant processed their perception of this process and noted,

I understand my identity as a professional, and how the client receives me as a therapist and the power dynamics at play there. Just because a client may say, to some extent, like, “oh, yeah, I guess I'm comfortable with touch”, I always take into consideration that there might be pressure to say yes, because of the power dynamics in the therapy room.

One participant discussed their awareness of the privileges their physical identity holds. They described,

…they [client] know the person of the therapist because that's what they see, and I represent a lot of groups that aren't warm and comforting and, you know. I think about what those groups I represent when just walking into the room. Right? So, I always have to be a little bit more mindful of that… and I have to be knowledgeable about how much power I have when we're talking about touch.

These exemplars highlight how therapeutic touch cannot be fully understood without accounting for how our physical attributes are constituted in the social world by skin color, shape, size, movement, femininity, masculinity, and ability.

#### 4.5.3 Therapeutic Setting

In addition to discussing the ongoing consideration of power dynamics, there was a conversation surrounding the evolving view of the therapeutic setting and its impact on the use or consideration of touch in therapy. One participant discussed their perceptions of touch when working in a correctional facility and shared,

…that was just like, not a natural thing for me to do that. I just had to keep those boundaries… in residential treatment, it's just so important, because you're in their living space and things of that nature…and if someone was like, ‘you need to follow the model and you need to do touching’, it would be so incongruent.

Participants also processed how some settings where clinicians work, touch may be more common or expected, creating different standards for the use of touch and Satir's model. One participant shared that it felt more normalized or congruent in the medical space they worked in and noted,

I've been practicing in primary care as a medical family therapist. People come into a primary care office expecting to be touched, you know, or examined or whatever, and plus the room I was in was a broom closet. So, we were already kind of on top of one another. So, a closeness or a gentle slight touch wouldn't be out of the norm.

Participants also highlighted the age group of their clients, in addition to the setting. Another clinician described their work with older adults in a retirement home where helping them move through spaces was normal creating an easier avenue to incorporate touch. They described,

So, I have to kind of be mindful of that [therapeutic setting] you know I do a lot of work with older adults, I touch them all day, right? Just, you know, even like a rub on the arm, or, you know, embrace or something at the end…the dynamics are different, you know, with those populations.

Understanding your clinical position in addition to critically attuning to the therapeutic setting is essential when considering the use of touch. In addition to being mindful of the environment, the therapist must explore their conceptualization of the model as they work to refine their individual approach and perspective of the model in their current context.

#### 4.5.4 Model Evolution

In early videos of Virginia Satir doing therapy, her use of touch was vastly different from what current clinicians might consider utilizing in practice. She was bold, gentle, and assertive at the same time, with an engaging presence that seemed to disarm clients and keep them engaged. Although profound, the acceptability of her style of touch may be attributed to different contextual factors related to her presence and societal perceptions surrounding personal autonomy at the time. Participants in this study noted how the conceptualizations of touch in this model have evolved over time for various reasons. One shared,

Touch is a very tricky thing these days, it wasn't as tricky in Virginia's day as it is now. You can still do it now. But you know, it's really important to get permission and ask if it's okay with the person because you never know when it might not be. You want to respect that because the first step is always trying to build safety between you and the other person.

Another participant similarly highlighted,

Yeah well, these days, you know, touching is sort of out of the picture with most other models…I mean, it's still in the picture with Satir but you have to be very careful about touching clients. There's laws and, you know…but I think touches are very important part of the process.

Clinicians in this study emphasized the differences between historical and modern‐day applications of this model in addition to offering a reminder that this model can be personalized based on the comfort and personhood of the therapist. A participant outlined,

I think that… people felt very safe with her, and she used touch in such a healthy way. I don't think we have to use it in the same way. I take it, by their [the client] cue you know, I don't impose it. But it's such a way of kind of deep connection, and touch can feel very affirming, but I don't use it throughout the entire session, like she would.

Regardless of if the therapists in this study utilized touch, there was an overwhelming message hoping that this aspect of the model did not dissuade present‐day clinicians from using this approach.

I've never met Satir in person, but I believe that if somebody said, like, “oh, well, I can't practice the model, because I'm not comfortable with touch”. I think Satir would be aghast at that. Because she was very much like, ‘hey, here are the boundaries of this model’. What's exceptionally important as a therapist, is that you are you, and if touch between you and a client is not your thing, like, fine make it you! Right? That doesn't mean that it's doesn't count. I think she would maybe have other thoughts if you said, like, ‘well, I don't want to facilitate touch in family systems’, then I think she might be like, ‘alright, now I got a bone to pick’. I've seen clinicians, especially young clinicians that that don't have a very firm grasp, of Satir get kind of pushed away from that very narrow definition of what Satir is.

The Human Validation Process Model offers unique methods to connect and support clients throughout their time in therapy. It is essential that clinicians understand the multidimensional aspects of this approach for critical conscious application. Doing so includes recognizing how aspects of the model exist in modern‐day systemic contexts.

## 5 Discussion

The present study explored if, when, and how therapists who employ the Human Validation Process Model, use or conceptualize touch in their clinical work. Establishing a clearer understanding of this intervention in the context of the model can support practitioners in understanding its potential utility. Historically, there have been mixed perspectives about the use of touch in therapy, in part due to the potential for harm if used unethically or without clear boundaries in place. Satir emphasized the value of touch as a method to soothe or intensify a moment in therapy, but did not clearly outline alternatives or other important factors for therapists to consider.

This study sought to explore these gaps by interviewing 15 clinicians utilizing the HVPM. The analysis resulted in five overarching themes: (1) establishing a congruent connection, (2) orchestrating touch, (3) handling with care, (4) self of the experiential therapist, and (5) evolving contemporary considerations. The results of this study provide updated commentary and nuance surrounding the ways clinicians utilizing the HVPM might “make contact” with their clients. In the context of the model, “making contact” is the experience of creating a genuine connection with the client where the therapist understands their verbal and non‐verbal cues (Satir 1976). Participants in the study emphasized the importance of connecting with clients and, at times, using touch to do so. They described the value of using touch to soothe clients, after asking for consent. This was consistent with similar research that found that clinicians often feel naturally inclined to offer a hug or a form of closeness when witnessing a client who is distraught (Harrison et al. 2012). In addition, participants emphasized that touch is implemented through commonplace greetings like handshakes or hugs. For some, this felt within their boundaries, and others were overt in clarifying with clients if they did not engage in touch‐oriented greetings. Clinicians must create a culture in therapy where clients feel they can advocate for themselves should they wish to decline a touch gesture.

In addition, participants noted feeling empowered by orchestrating touch among clients and utilizing this intervention indirectly. Some participants highlighted the impact of sitting close to or leaning in when clients were discussing a heavier topic to help ground themselves and the client in the present moment. Interestingly, therapists who did not feel comfortable touching clients directly shared that they also found this intervention useful. For example, touch‐averse therapists could direct their clients to hold hands when communicating or point out when clients organically touch each other in session. Doing so allowed clinicians to support clients in having a new experience in therapy through touch, which is aligned with the core tenets of the HVPM, while also maintaining congruence with their professional boundaries.

Although there is limited guidance on the use of touch by psychotherapy ethical codes (Calmes et al. 2013), participants expressed that they are actively considering the complexities of touch through an ethical and trauma‐informed lens. Therapists in this study reported that the lack of formal regulation heightens the responsibility for clinicians to engage in deliberate, context‐specific decision‐making when using touch in this model. Specifically, participants highlighted that touch can be healing but also harmful, especially for clients with trauma histories. These findings echo larger ethical conversations regarding moving away from the binary that touch is “always bad” or “always good” and move toward nuanced reasoning (Zur 2007). Findings related to consent for touch as a dynamic process align with trauma‐informed care principles of client agency, safety, and empowerment (Substance Abuse and Mental Health Services Administration SAMHSA 2014). Consistent with prior research, therapists in the current study reported that decisions to use touch were often made instinctually (Harrison et al. 2012). Rather than taking an abstinence‐only approach, we must attune to the lived experiences of our clients to create clinically congruent, trauma‐informed, and culturally relevant treatment plans. Together, we can use their voices to shape research‐informed practices and systemic supervision approaches to support the ethical use of touch in therapy.

Participants in this study recognized the importance of understanding their own personhood and how this might play a part in their conceptualization of touch in the HVPM. They reported that they aimed to understand this topic through a critical lens that took their own and their client's identity into account. Along with client identities, it is crucial to consider how bodies shape therapeutic touch based on how they are socially read, physiologically regulated, and experienced. For example, gender‐diverse clients may experience heightened anxiety around the use of touch due to misrecognition of their identity or social stigma. Similarly, neurodiverse clients may have a different sensory experience with touch that is not captured in universal or research‐based approaches to touch in therapy. Further, the appearance of bodies intersects with power and the meaning of touch. For example, white bodies have historically enacted physical violence against Black bodies (Menakem 2017). We must reflect more on how our embodied experiences impact our use and receipt of touch in the world. Judgments on how clients and therapists are perceived in relation to age, race, and gender impact the safety, accessibility, and impact of touch.

Extant literature has emphasized how the therapist's self‐awareness and healing are critical for effective training and development as a practitioner (Aponte 2022). It is vital that clinicians understand what touch might mean coming from someone with their identity, in addition to how it might be received by another based on their identity and prior experiences. Understanding touch in the current context is vital, in addition to ensuring there is an understanding of the historical significance of touch in the context of the client's culture and identity. For example, when working with female‐identifying clients, ensuring there is awareness of social movements related to bodily autonomy, such as the #MeToo movement. There is extensive evidence documenting the prevalence of gender‐based harm and the profound impacts these experiences have on client boundaries and safety, specifically for female bodies being touched by male bodies (Wexler and Sweet 2021). Social movements such as #MeToo and Times Up have further illuminated the critical intersection of sex and power (Gill and Orgad 2018). Considering what these experiences might mean in the context of a client's trauma history, the intersections of the therapist and client identities must be held with intentionality and care. Various aspects of identity may be more salient for some when considering utilizing touch, which deems the ongoing development and evolution of critical consciousness all the more important.

The use of touch in therapy is a nuanced and historically controversial intervention that requires careful attention to identity, power, and cultural context. This model calls for touch that is carefully considered by the therapist and only implemented by clinicians who are aware of their ethical responsibilities and clearly understand its utility in the context of the HVPM. Research has discussed the importance of understanding the therapist's social positioning (Watts‐Jones 2010) and attuning the client's intersectional identity (PettyJohn et al. 2020), but never clearly as it relates to touch in the context of this model.

## 6 Conclusion

The present study offers a timely contribution to the limited body of research examining the use of touch within Satir's HVPM. This study utilized a phenomenological framework to explore the experiences of experiential therapists working to conceptualize and implement touch in therapy. Through a reflexive thematic analysis of experiential therapists' narratives, the findings illustrate the complexity, intentionality, and nuance that surround the use of touch in psychotherapy work. While Satir emphasized the value of touch, this study provides clarity on how modern clinicians interpret, adapt, and navigate using this intervention through a trauma‐informed and identity‐aware lens. Specifically, clinicians describe using touch both directly and indirectly, attending purposefully to consent, context, and safety. Further, participants emphasized shifting away from a binary perspective of touch to one that centers relational attunement and self‐reflection.

Importantly, these findings breathe new life into the model by demonstrating how its core values of contact, empathy, and human connection can still be honored even when certain techniques may not feel congruent for every therapist. As societal norms and expectations continue to evolve, it is imperative for us to review and adapt our interventions to meet the needs of modern clients. Clinicians are called to reimagine the possibilities of using interventions in this model instead of abandoning it.

The current study possesses a variety of strengths and contributions to current literature on systemic therapy models. Results of this study present insights into the use and utility of touch within the Human Validation Process model. This is one of the first to focus solely on the use of touch in the context of this model, in addition to providing insights into *how* a therapist might use or incorporate touch in their practice should physical touch feel clinically incongruent. In addition, alternatives for incorporating indirect touch in therapy are presented, including manipulating the space, facilitating the connection between clients, and highlighting natural client touch. Lastly, this study provides insights from therapists about how they consider their own identity and personhood before incorporating touch in their practice.

Despite the strengths of this study, there are also limitations that should be considered for future research to build upon these findings. Considering this study concentrated on a specific intervention in the HVPM with a small sample size, the generalizability of these findings is limited. In addition, participants in this study were all located in the United States. The HVPM gained popularity in various countries abroad, where views of touch may differ. Future research should aim to include international perspectives of this model and intervention to broaden our perspective of how touch may be supportive for clients from non‐Westernized cultures. Moreover, participants in this study practiced in a variety of settings where touch may be perceived differently (i.e., primary care settings). Conceptualizations of this intervention may evolve to meet the needs of clients in settings where touch is often expected. Lastly, this study did not overtly explore how touch might be used and interpreted differently when working with children. Participants in this study primarily worked with adult systems, leaving room for further exploration of how working with children might require additional considerations for practice.

In sum, the findings of this study affirm that “making contact” can manifest in a multitude of ways, both physical and nonphysical, as the core of the HVPM is an ethical, intentional goal of human connection. Touch interventions have been found to have physical and mental health benefits, especially in clinical populations (Packheiser et al. 2024). Our study highlights the ongoing need for dialog, research, and training that supports the evolution of therapy models and trauma‐informed care. These examinations ensure that our founding models remain relevant to contemporary clinical practice and grounded in the transformative principles that define them. In addition, we are able to continually assess and advocate for the safety and beneficence in clinical practice.
